# Workplace Violence against Hospital Workers during the COVID-19 Pandemic in Israel: Implications for Public Health

**DOI:** 10.3390/ijerph19084659

**Published:** 2022-04-12

**Authors:** Keren Dopelt, Nadav Davidovitch, Anna Stupak, Rachel Ben Ayun, Anna Lev Eltsufin, Chezy Levy

**Affiliations:** 1 Department of Public Health, Ashkelon Academic College, Ben Tzvi St. 12, Ashkelon 78211, Israel; dopelt@bgu.ac.il (K.D.); an25122006@edu.aac.ac.il (A.S.); rclbnzio@edu.aac.ac.il (R.B.A.); anhlbalz@edu.aac.ac.il (A.L.E.); 2 Department of Health Policy and Management, School of Public Health, Faculty of Health Science, Ben Gurion University of the Negev, P.O. Box 653, Beer Sheva 84105, Israel; 3 Barzilai University Medical Centre, Hahistadrout St. 2, Ashkelon 78306, Israel; chezyl@bmc.gov.il

**Keywords:** healthcare workers, workplace violence, hospital, COVID-19, Israel

## Abstract

Workplace violence (WPV) against healthcare workers, a serious public health problem with profound implications, has worsened during the COVID-19 pandemic. This study examined the incidence of different types of WPV in a public hospital in Israel during the pandemic and analyzes the factors associated with its occurrence. A cross-sectional study was performed via an online questionnaire with 486 workers at a government hospital in Israel. Data were collected about sociodemographic and occupational characteristics, exposure to different forms of WPV over the preceding six months, and the responsibility and reasons for WPV from the workers’ perspective. Approximately 71% of respondents were exposed to WPV and 64% perceived that WPV escalated during the pandemic. The prevalence of verbal/psychological and physical WPV were 69 and 11%, respectively. The main reason for WPV was frustration over long wait times (70%). The escalation during the pandemic can be attributed to patients’ or relatives’ anxiety and mental states following the onset of the COVID-19 pandemic (72%), an increase in waiting time since the pandemic began (54%), lack of hospital resources to care for everyone (45%), and the inability to visit critically ill relatives with COVID-19 (44%). Increased exposure to WPV was attributed to lower seniority, working in emergency or internal departments, and being a nurse or a doctor. The findings raise an urgent need to develop strategies to reduce WPV in hospitals at all levels: national, organizational, and individual. Further research could focus on the effectiveness of innovative strategies and interventions to prevent violence against healthcare workers.

## 1. Introduction

Workplace violence (WPV) in healthcare settings, with its profound implications, has been acknowledged as a significant public health concern [1]. WPV includes threats, verbal or physical abuse, sexual harassment, shaming, property damage, beatings, and bullying [2]. It can cause physical and psychological harm, job dissatisfaction, anger, shame, anxiety, sadness, depression, insomnia, burnout, and increased turnover [3,4], resulting in abandoning the profession [5], in addition to the quality of the healthcare services provided and increased healthcare costs [6].

During the first wave of the COVID-19 pandemic, healthcare workers were lauded as heroes [7], but patients (and the public) no longer express the same appreciation. Indeed, for over a decade, professionals have been noting increased violence against healthcare workers, even in industrialized countries [8], a phenomenon apparently intensified by the pandemic [9,10,11,12,13]. Devi indicated that violence often worsens during emergencies [9].

Various factors may be driving WPV against healthcare workers during the current pandemic. First, healthcare workers were accused of spreading the disease [11]. Second, inadequate resources (ICU beds, oxygen tanks, ventilators) to admit or treat patients with COVID-19 generated anger [14]. Third, misinformation about the COVID-19 pandemic, especially when too much information, including false or misleading information in digital and physical environments during a disease outbreak (defined also as ‘infodemics’), led to panic, anxiety, and deep mistrust [12]. Fourth, the spread of fake news, coupled with religious conservatism and ignorance, sometimes added to a mistrust of science, the pharmaceutical industry, or the political system, and resulted in the diffusion of conspiracy theories (e.g., injection of nanochips in the guise of a COVID-19 vaccine) [15]. Fifth, bureaucracy, long waiting periods, inappropriate waiting areas, and lack of communication with healthcare workers were problems before the pandemic that were exacerbated as the pandemic progressed, with pandemic fatigue and frequent changes in pandemic regulations [8].

Pompeii et al.’s systematic review reported that between 2 and 32%, and 22 and 90% of hospital workers experienced physical violence and verbal abuse, respectively [16]. Liu et al.’s meta-analysis found that between 27 and 45% of nurses worldwide have experienced physical and non-physical violence [17]. WPV against healthcare workers (mainly physicians and nurses) was also reported in studies conducted in Switzerland (50%) [18], Israel (58%) [19], Saudi Arabia (58%) [20], and Australia (71%) [21]. 

Byon et al. found that during a five-month period early in the COVID-19 pandemic in the United States, 44 and 68% of registered nurses reported experiencing physical violence and verbal abuse, respectively [22]. Moreover, approximately one-fifth of participants said they experienced more violence during the pandemic than before. A survey among physicians and nurses in two public hospitals in southern Egypt revealed that during the six months preceding the COVID-19 pandemic, 43 and 10% reported exposure to psychological and physical WPV, respectively. The patients’ relatives were the perpetrators in 75 and 90% of cases of psychological WPV and physical WPV, respectively [2]. In Brazil, Bitencourt et al. found that 48% of participants reported violence against health professionals during the pandemic [13]. In Pakistan, 38% experienced violence in the preceding six months, 34% experienced verbal violence, and 7% physical violence [23]. In a government hospital in Jordan, where patients’ relatives were the principal perpetrators in most incidents, 66% of surveyed healthcare workers reported exposure to WPV, mainly verbal violence (52%) [24]. Nurses and physicians are more vulnerable to WPV than other professions working in hospitals [8,13,17,20,21]. Moreover, studies show that emergency department workers are at a higher risk for violence, compared to workers in other health settings [3,8,17,18,23].

WPV against healthcare workers is a complicated problem [25], with serious implications for workers and the healthcare system in general [3,4,6,8]. It frequently occurs, with consequences that can affect their psychosocial well-being and the assistance given to their patients and families [13]. The reasons people attack healthcare staff during health emergencies are many (e.g., fear, panic, misinformation, mistrust, etc.) and vary according to local contexts [14]. The alarming increase in WPV against healthcare workers during the COVID-19 pandemic highlights the urgent need to understand, prevent, and address these events, and identify the predictors of violence, especially during pandemics. While high levels of psychological and physical WPV toward hospital workers during the COVID-19 pandemic has been studied in various countries, the incidence of forms of WPV in Israeli hospitals during this period has not yet been assessed. This study aims to examine the incidence of different types of WPV in a public hospital in Israel during the COVID-19 pandemic and analyze the factors associated with its occurrence. The research hypothesis is that emergency department workers will be more exposed to violence relative to other departments. Moreover, nurses and physicians will be more exposed to violence relative to other professions.

## 2. Materials and Methods

A cross-sectional study was conducted among healthcare workers at the Barzilai University Medical Centre, a government hospital with 567 beds and an additional 60 day-patient beds, in November and December of 2021. The study was approved by the Ashkelon Academic College Ethics Committee (approval #34-2021) and hospital management.

### 2.1. Procedure

A link to the survey was sent to all Barzilai University Medical Centre workers via email from the human resources department on 10 November 2021. Every two weeks, a reminder was sent to increase the response rate, with a total of three reminders. The survey closed on 29 December 2021. Within the convenience sample, 486 staff members from all sectors who completed at least 95% of the survey were included in the study (26% response rate). A comparison between sample characteristics (sex, age, and profession) and human resources department data revealed that the characteristics of non-respondents and respondents were not statistically different.

### 2.2. Study Tools

The online survey comprised 18 questions taken from the Israeli Medical Association (IMA) workplace-violence questionnaire (see Appendix A for the English translation of the Hebrew survey). For validation purposes, the questionnaire was given to two physicians, three nurses, two social workers, and two medical secretaries from various departments, genders, and ethnic origins from another hospital for evaluation. Five questions were corrected based on written comments they made. After the validation process, the inter-rater reliability testing revealed generally high reliability for ratings for the different parts of the questionnaire. The anonymous questionnaire included several sections:

1. Demographic and occupational details: nine questions regarding gender, marital status, religion, age, country of birth, profession, seniority, department, and work in the coronavirus ward.
2. Exposure to seven different forms of WPV in the last preceding months: verbal violence; verbal threats; passive-aggressive behavior (intrusion into personal space, facial expressions); destruction of property (throwing a chair, breaking an instrument); physical violence; sexual harassment; and internet ‘shaming’ (such as social networks). Participants were asked to indicate one of the following responses: (a) I did not experience WPV; (b) I experienced WPV from a patient; (c) I experienced WPV from a patient’s companion. A new measure was produced to compare the rate of WPV experience between groups—‘exposure to violence’—defined as being exposed to at least one event in the preceding six months.
3. Reasons for violence in the hospital in their judgment: participants could choose from eight given reasons and an option to indicate other reasons, such as dissatisfaction with the attitude/treatment of the staff, the effects of alcohol, long wait times, or uncomfortable physical conditions.
4. Contribution to violent incidents in the hospital: participants were asked to indicate the extent to which each of the following contributed to the incident: patient behavior, patient’s companion’s behavior, participant behavior, and medical staff behavior. This was measured on a Likert Scale ranging from (1) not at all to (5) to a very great extent.
5. Unique reasons for violence during the pandemic: two questions were asked: Do you think the number of cases of violence against hospital workers: (1) has decreased compared to the period before the COVID-19 outbreak; (2) has remained the same; (3) has increased; (4) I don’t know. The second was: What do you think could be causing an increase in incidents of hospital violence during the COVID-19 pandemic? More than one answer could be marked: patients’ or relatives’ anxiety and mental state following COVID-19; lack of hospital resources to take care of everyone, etc.
6. Responses to violence: four questions measured on a five-point scale were asked: Were you absent from work due to a violent incident you experienced?; Did you turn to emotional support due to a violent incident you experienced?; Do you feel the hospital management tries to prevent WPV against the hospital workers? There was one open-ended question: What do you think can be done to prevent WPV against hospital staff?

### 2.3. Data Analysis

The data were processed anonymously using SPSS v.26 software (IBM, Armonk, NY, USA). To adjust for sampling biases and ensure that the sample was representative, we compared and found no significant differences between respondents and non-respondents for sex, age, profession, and seniority. Exploratory data analysis demonstrated that the data were normally distributed, and parametric statistical tests were used. We compared survey responses by testing differences between professions and departments using chi-squared tests. Finally, logistic regression was conducted to predict the odds ratio for being exposed to WPV. All reported *p*-values are based on two-sided tests and were considered significant below 0.05.

## 3. Results

### 3.1. Sample Characteristics

The hospital employs around 1900 staff members, including approximately 300 physicians and 800 nurses. Table 1 shows the sample’s characteristics. As Table 1 illustrates, most respondents were female (consistent with the actual ratio of male to female hospital workers), partnered, and Israeli-born. Of them, 29% were working during the survey or had worked before the study in coronavirus wards. The most common profession was nursing (42%, consistent with the actual ratio of nurses to other hospital workers). Physicians accounted for 21% of the total sample (slightly higher than their proportion of total hospital staff—16%). The age of the participants ranged from 21 to 73 (average 42 ± 11.98), and the seniority ranged from 0.5–47 years (average 15 ± 2.34).

### 3.2. Exposure to Different Forms of WPV

Table 2 shows the distribution of the exposure to various forms of WPV as indicated by participants.

Quantifying the number of forms of violence that each participant experienced, we found that 32% (*n* = 154) were exposed to one to two forms of violence, 31% (*n* = 151) were exposed to three to four forms of violence, and 8% (*n* = 41) were exposed to five to seven forms of violence. Overall, 29% (*n* = 140) were not exposed to WPV, while 71% (*n* = 346) were exposed to at least one event in the six months preceding the survey.

Chi-squared tests revealed significant differences between professions (χ^2^ = 28.91, *p* < 0.001), departments (χ^2^ = 40.50, *p* < 0.001), and work in a coronavirus ward (χ^2^ = 19.73, *p* < 0.001). The nursing profession experienced the highest level of exposure to at least one type of violence, followed by physicians and others (81, 78, and 57%, respectively). Almost all emergency department workers were exposed to violence, followed by general ward workers and, finally, others (93, 85, and 61%, respectively). Of those who work/have worked in the coronavirus department, 86% were exposed to at least one type of violence, compared to 65% of those who had never worked in this department. That is, the research hypotheses were confirmed. No significant differences were found between gender, religion, being in a relationship, and country of birth.

### 3.3. Reasons for WPV against Hospital Workers

The main reason for WPV against the hospital workers participants indicated was long waiting times (70%), the patient/companions arrived for treatment already angry (59%), dissatisfaction with the attitude of the treating staff (57%), bureaucracy (49%), dissatisfaction with the treatment (48%), uncomfortable physical conditions (36%), the effects of alcohol/medications/drugs (33%), racism (29%), and communication problems (20%).

After the participants who marked ‘I don’t know’ were removed (109 participants), 64% reported a perception that the number of violent incidents had increased compared to the period before the COVID-19 outbreak, 28% thought it had remained the same, and the others felt it had decreased (8%). They were asked what they believed could have caused an increase in the number of hospital violence incidents during the pandemic. The leading cause was patients’ or relatives’ anxiety and mental state following COVID-19 (72%), an increase in waiting time since the pandemic began (54%), lack of hospital resources to take care of everyone (45%), inability to visit a critically ill relative who had COVID-19 (44%), and the enforcement of the one companion per patient limit (40%).

### 3.4. Contribution to the Violent Incidents in the Hospital

Table 3 shows the distribution of the contribution to workplace as evaluated by the participants.

Participants estimated that the patients and companions contributed the most to hospital violence incidents. They nonetheless recognized the responsibility of the staff and their own contribution to violent events.

### 3.5. Logistic Regression Model

Logistic regression was performed to examine the influence of the department, profession, and seniority in terms of being subjected to WPV. The regression model was significant (χ^2^ = 79.25, *p* < 0.001), explaining 23% of the variance in exposure to WPV (Nagelkerke R^2^). It was found that seniority lowers the chance of being exposed to WPV; working in the emergency department increases the chance of being subjected to WPV by 630%; working in the general ward increases the chance by 105%; being a nurse increases the chance by 258%; and being a physician increases the chance by 229%. Table 4 shows the odds ratio of being exposed to WPV.

### 3.6. Responses to Violence

Among the participants, 5% were absent from work following a violent incident, and a similar percentage turned to emotional assistance. About a third (31%) felt that hospital management tried to deal with WPV to a small extent, 30% to a moderate extent, and the rest thought that hospital management tried considerably to deal with this phenomenon. The open-ended question ‘What do you think can be done to prevent WPV against hospital staff?’ was answered by 234 participants, with 36% answering additional security and about a third (32%) answering better communication and humane treatment, patience, improved service, and providing explanations to lower stress and anxiety. Nearly 19% indicated that more staff should be added to reduce waiting times and the resulting frustration that would increase patients’ and families’ patience and minimize staff burnout. Sixteen percent argued that penalties should be more severe in order to deter violence. A tenth thought that waiting times and bureaucracy should be reduced. Nine percent claimed that waiting and hospitalization conditions should be improved; management should support staff; the general population should be educated to respect medical staff; and visiting hours should be limited.

## 4. Discussion

Our study was conducted shortly after the fourth wave of the pandemic (mainly Delta variant) and before the start of the fifth wave (mainly Omicron); thus, answers mostly reflected what happened during the fourth wave when vaccines were already available, but the virus spread faster than in previous waves, and both the public and healthcare workers suffered from pandemic fatigue. As found by Byon et al., participants estimated that incidents of violence increased during the pandemic (64%) [22]; more than two-thirds of the participants (71%) reported having experienced WPV six months before the survey. Healthcare workers are sixteen times more likely to suffer from WPV than other professions, and hospitals are the main settings where it happens [26]. It is possible that the combination of the profession (healthcare workers), the setting (hospital), and the escalation during the COVID-19 pandemic led to such a high incidence of WPV. Healthcare workers who experience WPV are more prone to job dissatisfaction, burnout, and mental health problems [1,3,4,27,28,29,30]. In the context of the pandemic, exposure to WPV may exacerbate existing stress and burnout that hospital workers face [7].

Previous studies conducted during the COVID-19 pandemic used several tools and definitions of WPV among various healthcare professions and different assessment durations. Therefore, it is hard to compare our findings to previous results. Nevertheless, it seems that the prevalence of WPV found in the current study is higher than that observed in other countries [2,13,23,24,31]. Participants estimated that the primary contributor to violence was the behavior of patients’ companions, as found in previous studies [2,24,32]. However, we found similar rates of violence perpetrated by patients and by relatives (Table 2).

Nurses experienced the highest WPV (81%) followed by physicians (78%) (compared to 57% among others), as both professions are on the front line and involved in direct patient care. These findings are consistent with previous studies [17,20,31,32,33,34]. Almost all emergency department workers were exposed to violence, followed by general ward workers and others (93, 85, and 61%, respectively). Emergency department workers are at high risk for WPV, compared to those working in other healthcare settings [28,31,35,36]. In a meta-analysis, D’Ettorre et al. found that 32 studies showed a prevalence of between 24 and 89% of healthcare workers in emergency departments having been victims of WPV by a patient at some stage in the preceding 12 months [28]. According to Alharbi et al., 40% of those who experienced WPV did not report it to the hospital management, mainly because they perceived it ‘would not change anything’ [32].

Underreporting WPV incidents is a global problem [32,37], so we can judiciously assume that our findings have just begun to explore the depth of the problem. The logistical regression reinforced these findings. It also reinforced the idea that workers with higher seniority are less exposed to WPV, consistent with several other studies [26,38,39]. Studies have shown that professional experience improves the ability to manage conflict situations with angry patients [10]. In a comprehensive review, Civilotti et al. demonstrated a high prevalence of WPV against healthcare workers in Italy, especially in emergency departments and among nurses and physicians [34].

Long wait times, dissatisfaction, bureaucracy, and alcohol/medications/drugs effects were frequent determinants of WPV, a finding consistent with the WPV literature [28,36,40]. The leading causes for the deterioration in WPV during the pandemic were anxiety and poor mental state following COVID-19 [12], increased waiting times [8], and a lack of hospital resources [14].

To prevent WPV in the hospital, participants suggested improving the hospital’s security system. Moreover, better communication skills and increasing the workforce in all professions were seen as ways to lower violence by reducing long wait times and patients’ and companions’ frustration. This would lead to less pressure on staff, resulting in less burnout and more patience towards the patients, supporting the finding that participants took responsibility, acknowledging that general staff behavior and their own behavior contributed to violence. Wu et al. demonstrated the association between workload and WPV, with high-stress situations and daily overload associated with WPV [40]. The researchers explained that high job demand and overload lead to poor quality care and, consequently, to frustrated patients, which is one of the leading causes of WPV.

WPV creates a vicious cycle, affecting the attitudes of healthcare workers, which results in a higher probability of new violent incidents [13]. Thus, WPV must be prevented and condemned in the interests of creating a safer environment in hospitals by means of a ‘zero tolerance’ attitude concerning violence against healthcare workers.

### Study Limitations

Several limitations should be noted. First, because the study is a cross-sectional study, inferences of causality cannot be made. Second, to collect data, we used self-reporting measures, which can be biased due to selection bias or social desirability. However, anonymity can mitigate these biases. Finally, the study was conducted in one hospital, which could affect the generalizability of the study’s findings. Future studies conducted in numerous hospitals could replicate and support the current results.

## 5. Conclusions

This study contributes to raising awareness of the need to mitigate WPV against healthcare workers in Israel and globally. The need for such awareness and structural changes has, unfortunately, increased during the current public health emergency due to the rise of the COVID-19 information epidemic, ‘infodemics’. This demonstrates how unlimited access to information can affect behaviors during a health crisis. This led to threats to healthcare officials and field workers, and overwhelmed public healthcare systems, underfunded due to budget cuts and poor priority management. Political instability and growing mistrust among all stakeholders are also important factors to be considered. Unfortunately, violence against healthcare workers did not start with COVID-19, but has only escalated since the onset of the pandemic, along with higher workplace demands, workload, and pandemic-induced anxiety. Violent events aggravate these situations. As a result, national, organizational, and individual interventions are needed. For example, governments should promote and strongly enforce harsher legislation, including penalties for aggressors. The police and the courts pay little attention to the phenomenon and attackers are rarely penalized. Moreover, additional resources should be allocated to increase the number of hospital staff to alleviate workload and waiting times. Hospital leadership should be committed to ensuring a safer environment for workers and improving security arrangements, especially in departments prone to violence, like emergency departments. Hospitals need to invest in workshops and training for improving workers’ communication skills. Workers need to be trained to identify potential violence early on and thus prevent incidents of violence, especially given their own admission that staff behavior contributes to violent incidents. Staff can improve their capacity to provide empathetic and considerate care to reduce WPV. Additionally, the issue of trust and the political context should be taken into consideration when addressing WPV within specific local contexts. Further research could focus on the effectiveness of innovative strategies and structural interventions to prevent violence against healthcare workers.

## Figures and Tables

**Table 1:** Study sample characteristics (*n* = 486).

Characteristics	N	%
Male	146	30
Female	340	70
In a relationship	369	76
Place of birth:		
Israel	316	65
Former USSR	125	26
Other	45	9
Working/have worked in coronavirus ward	139	29
Role:		
Physician	100	21
Nurse	205	42
Other (management and housekeeping, computing, auxiliary staff, laboratory)	181	37
Most common departments:		
General	99	20
Emergency Department	80	16
Other (gynecology, cardiology, children, labs, management, etc.)	307	64

**Table 2:** Exposure to various forms of workplace violence.

Forms of WPV	Experienced from a Patient	Experienced from an Attendant	Experienced from a Patient or Attendant or Both
Verbal violence	45%	46%	63%
Passive-aggressive behavior	37%	38%	55%
Verbal threats	26%	28%	40%
Destruction of property in protest	12%	9%	17%
Physical violence	8%	6%	11%
Sexual harassment	7%	3%	9%
‘Shaming’ on the internet	4%	4%	6%

**Table 3:** Contribution to the violent incidents in the hospital.

Category	To a Small Extent (Answers 1 + 2)	To a Moderate Extent (Answer 3)	To a Very Great Extent (Answers 4 + 5)
Companion’s behavior	6%	15%	79%
Patient’s behavior	15%	22%	63%
Staff behavior	34%	23%	43%
Participant’s behavior	47%	19%	34%

**Table 4:** Logistic regression model to predict exposure to workplace violence.

Predictors	B	S.E.	Wald	df	Sig.	Exp (B)
Seniority	−0.031	0.009	10.760	1	0.001	0.969
Physician	1.190	0.326	13.284	1	0.000	3.287
Nurse	1.276	0.264	23.370	1	0.000	3.581
General ward	0.719	0.329	4.778	1	0.029	2.052
Emergency department	1.988	0.455	19.090	1	0.000	7.301
Constant	0.311	0.229	1.844	1	0.174	1.365

## Data Availability

The data that support the findings of this study are available from the first author upon request.

## Abbreviations

WPV	Workplace violence
COVID-19	coronavirus disease 2019

## Appendix A. Questionnaire

In the light of the many cases of violence against healthcare workers, this questionnaire is designed to study the level of exposure to violence among hospital workers. Filling out the questionnaire will take about five minutes and is voluntary and anonymous. Completing the questionnaire constitutes consent to participate in the survey.

For questions, please contact Dr. Keren Dopelt by email dopelt@bgu.ac.il.

Thank you for your cooperation!

1. Gender: (1) male; (2) female; (3) I don’t want to answer.

2. Marital status: (1) I am in a relationship; (2) I am not in a relationship.

3. Religion: (1) Jewish; (2) Muslim; (3) Christian; (4) Atheist; (5) other—_____.

4. Age: _______.

5. Country of birth: (1) Israel; (2) Former USSR; (3) other—__________.

6. Profession:______.

7. Seniority in the profession:_______ years.

8. Department:_____________.

9. Do you work in the coronavirus department? (1) yes; (2) I worked there during the previous waves (3) no.

10. Have you experienced violence of the following forms in the last six months?

**Forms of Workplace Violence**	**I Did Not Experience Workplace Violence**	**Experienced Workplace Violence from a Patient**	**Experienced Workplace Violence from an Attendant**
Verbal violence			
Verbal threats			
Passive aggressive behavior			
Destruction of property in protest			
Physical violence			
Sexual harassment			
‘Shaming’ on the internet			

11. In your opinion, what are the reasons for violent incidents in the hospital? (More than one answer can be marked): (1) dissatisfaction with the attitude of the staff; (2) dissatisfaction with the care of the treating staff; (3) the effects of alcohol/drugs; (4) the patient/their companions arrived at the hospital already angry; (5) racist perceptions; (6) long wait times; (7) uncomfortable physical conditions; (8) long bureaucratic processes; (9) language/communication problems; (10) other—___.

12. In your opinion, to what extent does each factor contribute to the incidence of violent events in the hospital?

	**(1) Not at All**	**(2) To a Small Extent**	**(3) To a Moderate Extent**	**(4) To a Great Extent**	**(5) To a Very Great Extent**
Patient behavior					
Companions’ behavior					
Your own behavior					
Behavior of the staff					

13. Do you think the number of violent incidents against hospital workers: (1) has decreased relative to the period before COVID-19; (2) has remained the same; (3) rose during the COVID-19 pandemic; (4) I do not know.

14. What do you think could be causing an increase in the number of violent incidents in the hospital during the COVID-19 pandemic? (More than one answer can be marked): (1) anxiety and poor mental state of patients and attendants following COVID-19; (2) the lack of hospital resources to take care of everyone because COVID-19 patients necessitate a large investment of financial resources and staff attention; (3) increased waiting time for treatment since COVID-19 outbreak; (4) limiting the number of attendants to one per patient; (5) the inability to visit sick relatives with COVID-19; (6) other:______.

15. Were you ever absent from work following an experience of workplace violence? (1) yes; (2) no; (3) I do not remember.

16. Did you seek emotional support due to a violent event you experienced? (1) yes, to _____; (2) no; (3) I have not experienced a violent event.

17. Do you feel that the hospital is dealing with violence against hospital staff? (1) not at all; (2) to a small extent; (3) to a moderate extent; (4) to a great extent; (5) to a very great extent.

18. What do you think can be done to prevent violent events against hospital staff? __________.
